# Highly Hydrophobic Organosilane-Functionalized Cellulose: A Promising Filler for Thermoplastic Composites

**DOI:** 10.3390/ma14082005

**Published:** 2021-04-16

**Authors:** Pavel Cerny, Petr Bartos, Pavel Kriz, Pavel Olsan, Petr Spatenka

**Affiliations:** 1 Department of Applied Physics and Technology, Faculty of Education, University of South Bohemia, Jeronymova 10, 371 15 Ceske Budejovice, Czech Republic; bartos@zf.jcu.cz (P.B.); kriz@pf.jcu.cz (P.K.); 2 Department of Agricultural, Transport and Handling Technology, Faculty of Agriculture, University of South Bohemia, Studentska 1668, 370 05 Ceske Budejovice, Czech Republic; olsan@zf.jcu.cz (P.O.); petr.spatenka@fs.cvut.cz (P.S.); 3 Department of Materials Engineering, Faculty of Mechanical Engineering, Czech Technical University in Prague, Technicka 4, 166 07 Prague, Czech Republic; 4 SurfaceTreat Plc., Jungmannova 695/42, 370 01 Ceske Budejovice, Czech Republic

**Keywords:** cellulose, hexamethyldisiloxane, hydrophobicity, plasma functionalization, polypropylene, thermoplastic composites, agglomeration

## Abstract

The aim of this work is to design and optimize the process of functionalization of cellulose fibers by organosilane functional groups using low-pressure microwave plasma discharge with hexamethyldisiloxane (HMDSO) precursor in order to prepare a compatible hydrophobic filler for composites with nonpolar thermoplastic matrices. Particular attention was paid to the study of agglomeration of cellulose fibers in the mixture with polypropylene. In our contribution, the dependence of the surface wettability on used process gas and treatment time was investigated. Scanning electron microscopy (SEM) and X-ray photoelectron spectroscopy (XPS) analyses were applied to characterize the surface morphology and chemical composition of the cellulose fibers. It was observed that the plasma treatment in oxygen process gas led to the functionalization of cellulose fibers by organosilane functional groups without degradation. In addition, the treated cellulose was highly hydrophobic with water contact angle up to 143°. The use of treated cellulose allowed to obtain a homogeneous mixture with polypropylene powder due to the significantly lower tendency of the functionalized cellulose fibers to agglomerate.

## 1. Introduction

Nowadays, environmental consciousness involving problems such as increasing emissions of harmful gases during incineration and increasing consumption of petroleum resources is growing. In addition, economic and environmental expectations of our society lead to widespread attention of engineers and scientists to the development of natural fiber reinforced polymer composites (NFPCs) [1,2,3,4,5,6,7,8].

NFPCs have been considered a promising composite materials for the last few decades with applications in many different fields of industry due to their outstanding performances in biodegradability, being lightweight, low cost, and having preferable mechanical properties [9,10,11,12,13,14,15,16,17,18,19,20]. Different types of NFPCs are used in various applications in the automotive industry by many automotive companies such as Audi Group, BMW, Daimler Chrysler, Ford, Mercedes, Opel, and Volkswagen. Moreover, NFPCs have also received great importance in the food, construction, and building industry, aerospace, and sports. Specifically, NFPCs are used for the production of bicycle frames, decking, door panels, furniture, food packaging, railroad sleepers, and window frames [21,22,23,24,25].

NFPCs are produced using natural fibers obtained from animal and vegetable sources [26]. All-natural cellulose fibers including cotton, coir, jute, sisal, etc., are extracted from various crops including bagasse, barley, corn, rice, sorghum, and wheat [27,28]. In order to meet the high demands of the industry, natural cellulose fibers have also been extracted from areca palm leafstalk, Saharan aloe vera cactus leaves, and Agave americana L. as reinforcement for NFPCs [29,30,31,32,33,34,35].

Natural fibers can be considered as a low-cost replacement for mineral and synthetic fibers. They are available all over the world, nontoxic, and renewable with low density, almost no carbon dioxide emissions and low abrasive effect on processing equipment [36,37,38]. On the other hand, there are several challenges that the development of NFPCs with nonpolar thermoplastic matrices must face [39]. Compared to mineral and synthetic fibers, natural fibers exhibit poor interfacial adhesion and have relatively low mechanical properties that vary depending on many factors, such as the shape, arrangement, density, etc., [40,41,42]. Moreover, mechanical properties of natural fibers can be affected by their undesirable moisture absorption leading to swelling [43,44,45].

In addition to low interfacial adhesion, composites with a non-polar matrix such as polypropylene (PP) and polar natural fibers present another problem related to the agglomeration of natural fibers due to forming hydrogen bonds between the hydrophilic fibers. Fibers tend to agglomerate into bundles and distribute unevenly in the mixture of the non-polar polymer matrix and the fiber reinforcement during the processing [46,47,48].

Above-mentioned drawbacks of natural fibers can be eliminated by suitable modification process including use of coupling agents, plasma discharges, mercerization, alkali treatment, peroxide treatment, acetylation treatment, and silane treatment [49,50,51,52,53]. Silane treatments are among the most widely used treatments for natural fibers. The advantage of silane coupling agents is the ability to interact with both the matrix and the fibers to form chemical bonds [54,55]. The chemical interaction between silane-based coupling agents and the fiber is mediated through the hydroxyl groups that are present on the surface of the natural fibers. The formed silanol is bound to the surface of the natural fiber by a covalent bond via hydroxyl groups or by hydrogen bonds [56]. Moreover, silane coupling agents can interact with maleated coupling agents (polyethylene or polypropylene-graft-maleic anhydride) to form covalent bonds [57,58,59,60,61]. Specifically, these are organosilane coupling agents with vinyl (VTS) and azide (ATS) functionality for use in NFPCs with polypropylene matrix [62,63].

Hexamethyldisiloxane (HMDSO) is one of the most widely used precursors of organosilane functional groups. HMDSO has found rich use in various plasma processes, including deposition and plasma polymerization [64], and it is currently increasingly used for the processes of functionalization of organic surfaces [65]. Plasma decomposition of HMDSO results in a variety of chemical structures. HMDSO molecules can be cleaved in plasma into hydrophobic fragments incorporated into the substrate surface, ensuring its high hydrophobicity [65,66]. Since organosilane-based treatments using HMDSO precursor often lead to hydrophobic and superhydrophobic surfaces, it appears to be promising not only for incorporation of organosilane functional groups but also for reducing the fiber agglomeration and moisture absorption [67,68,69,70].

The aim of this study is to design and optimize the cellulose fibers treatment process using low-pressure microwave plasma discharge with HMDSO precursor in order to prepare hydrophobic, non-moisturizing organosilane functionalized filler for thermoplastic composites with polypropylene matrix. Particular attention was paid to the study of agglomeration of cellulosic fibers in the mixture with polypropylene powder to make the compounding processing more efficient.

## 2. Materials and Methods

### 2.1. Materials

In our research we used the following materials and chemical agents:

- Cellulose fibers with a brand name of GW400 F (maximum humidity of 7%, minimum purity of 99.5%, fiber length 32–100 microns, fiber width 20–45 microns) were supplied by GreenCel Ltd. (Hencovce, Slovakia);
- HMDSO monomer (concentration 98–99%, molar mass 162.38 g∙mol^−1^) was purchased from Mach Chemikalie Ltd. (Ostrava, Czech Republic);
- UV and heat stabilized polypropylene powder with a brand name of Resinex RX 725 Natural (density 900 kg∙m^−3^, melt flow index at 230 °C 14 g/10 min, Vicat softening temperature 122 °C, Izod impact strength at 23 °C 21 kJ∙m^−2^, flexural modulus 1010 MPa) was purchased from Ravago Chemicals CZ Ltd. (Praha, Czech Republic).

### 2.2. Experimental Setup

**Figure 1:**
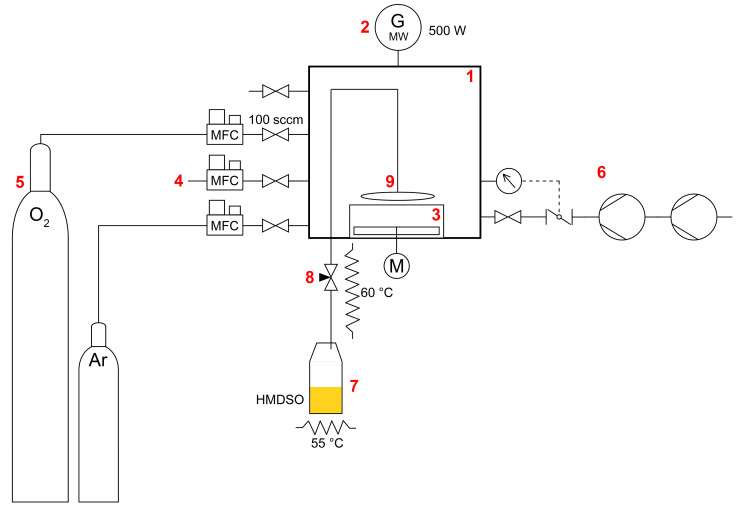
Scheme of used experimental setup: (**1**) vacuum chamber; (**2**) microwave power supply; (**3**) blender; (**4**) mass flow controllers; (**5**) process gas cylinders; (**6**) pumping system; (**7**) evaporator; (**8**) needle valve; (**9**) gas ring.

The scheme of the experimental setup is shown in Figure 1. Low-pressure microwave plasma discharge was generated in cubic vacuum chamber with a volume of 56 L made of stainless steel. Microwave power supply of total power up to 1 kW powered by a pulsed microwave power unit MNG 1K-08 supplied by Radan Ltd. (Barchov, Czech Republic) was set to 500 W microwave power. The cellulose fibers were mixed in a stainless-steel blender located inside the vacuum chamber. The blender with a base diameter of 20 cm and a height of 10 cm with a total volume of 3 dm^3^ was equipped with a horizontal propeller stirrer maintained at a fixed speed of 150 rpm.

System of two series-connected rotary oil pumps RV 100/1 supplied by Lavat Plc. (Chotutice, Czech Republic) and Adixen 2015SD supplied by Pfeiffer Vacuum Components & Solutions Ltd. (Asslar, Germany) was used to achieve the required pressure 100 Pa inside the vacuum chamber. The process gas flow rate of 100 sccm and the pressure inside the vacuum chamber were controlled by a set of mass flow controllers model F201-CV supplied by Bronkhorst High-TechH B.V. (Ruurlo, Netherlands) and regulated by butterfly valve of the pumping system controled by Adaptive Pressure Controler PM-3 supplied by VAT Vakuumventile Plc. (Sennwald, Switzerland), respectively.

An evaporator controled by a thermostatic control unit (maintaining an internal temperature of 55 °C) with a heated pipe (60 °C) connected to a vacuum chamber through a needle valve was used to evaporate the liquid precursor (HMDSO). The flow rate of the evaporated HMDSO was set to 5 sccm by needle valve. A gas ring device was used in order to evenly supply the precursor vapors to the treated substrate.

### 2.3. Characterization of Cellulose Fibers

Plasma treatment influences the wettability of cellulose fibres, chemical composition, and morphology of the surface. Therefore, water contact angle measurements (WCA), chemical composition analysis by the X-ray photoelectron spectroscopy method (XPS), and analysis of surface morfology based on emission scanning electron microscopy (SEM) were applied.

The water contact angle (WCA) was measured by the drop test method as described in [71] and also as reported in [72,73,74], in accordance with ISO 27448:2009. This method allows an easy evaluation of the characteristics of increasing hydrophobicity of the cellulose surface. Experimental setup was made of a portable computer-based instrument for contact angle measurement See System E supplied by Advex Instruments Ltd. (Brno, Czech Republic). The wettability of the cellulose surface was measured by initial WCA as well as the duration of absorption of water droplets (10 μL) settled on a compressed cellulose surface (dynamic WCA) in order to evaluate the surface hydrophobicity. The used water was distilled and colored with Acid Orange 7 organic dye for higher contrast.

The analysis of the chemical composition by the X-ray photoelectron spectroscopy method (XPS) was performed at the J. Heyrovsky Institute of Physical Chemistry of the Czech Academy of Sciences in Prague. The measurements were performed in a modified electron spectrometer ESCA 3 MkII under a vacuum of 10^−10^ mbar. Al Kα radiation (hʋ = 1686.6 eV) was used to excite the electrons. The transmission energy of the used hemispheric electron analyzer was 20 eV.

The morphology of the surface of untreated and treated cellulose fibers was characterized using JEOL JSM-7401F field emission scanning electron microscope (SEM) at the Laboratory of Electron Microscopy of the Institute of Parasitology (Czech Academy of Sciences, Ceske Budejovice, Czech Republic).

### 2.4. Processing of PP/Cellulose Mixtures

Before the mixing process, the cellulose was sieved to remove large agglomerates formed during production. Subsequently, polypropylene powder and cellulose fibers with a weight ratio of 80:20 were mixed in a cylindrical stainless steel blender located in the vacuum chamber previously used for mixing cellulose fibers during the low-pressure microwave plasma treatment process. The PP/cellulose mixtures were mixed for 15 min.

## 3. Results and Discussion

### 3.1. Optimization of Organosilane Functionalization Process

**Figure 2:**
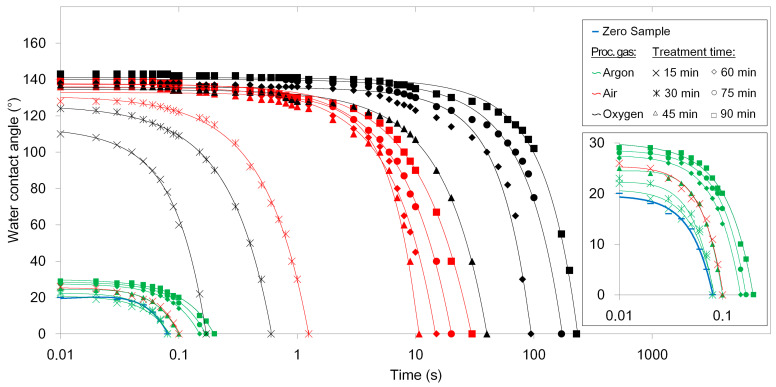
Dynamic water contact angle on cellulose samples for various process parameters.

In the first stage of our research, we studied the influence of process gas with HMDSO vapors and treatment time on WCA and the duration of droplet absorption (see variants in the Table 1 and Figure 2).

Consequently, the drop test has been used for the characterization of the surface hydrophobicity. Initial WCA has been estimated immediately after droplet settling on the cellulose surface. Afterwards, the time needed for complete absorption of the droplet into the material was measured and this value is denoted as duration of droplet absorption.

Whereas the samples treated in argon have almost the same wettability as the zero sample, samples treated in air and oxygen show clear dependence of both initial WCA and duration of droplet absorption on the treatment time. The longer the treatment time is, the longer it takes to absorb the water droplet into the material. In all three process gases the highest hydrophobicity of the surface has been estimated for the samples treated for 90 min.

Only a very slight increase in initial WCA and duration of droplet absorption can be observed for the samples treated in argon for longer time i.e., 45, 60, 75, and 90 min. Therefore, treatment in argon does not lead to a significant increase of the hydrophobicity of cellulose surface and even after the treatment for 90 min the cellulose surface remains strongly hydrophilic.

All samples treated in oxygen were highly hydrophobic and showed a slightly higher initial WCA compared to samples treated in air. A significant increase of the initial WCA can be well observed for samples treated longer than 15 min. Moreover, samples treated in oxygen also showed a significantly longer duration of droplet absorption, which is up to 229 s in the case of sample 18.

**Figure 3:**
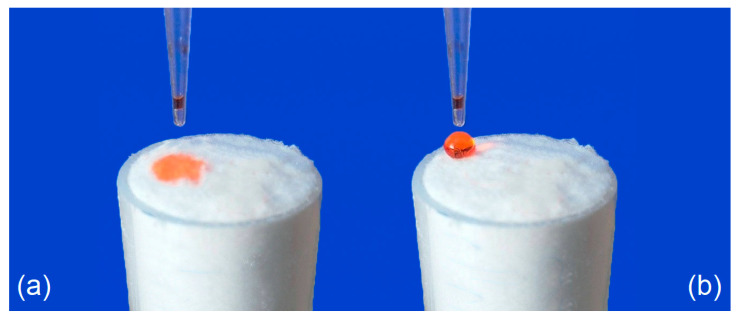
Comparison of surface wettability of compressed (**a**) untreated and (**b**) functionalized cellulose (sample 18) by optimized functionalization process.

The high hydrophobicity of the sample 18 compared to the zero sample is demonstrated in the Figure 3. Both photos have been captured immediately after settling of the water droplet.

Based on the data presented above, we conclude that the treatment of cellulose fibers in argon leads to only a slight increase in wettability. Conversely, hydrophobic cellulose was obtained by treatment in air and oxygen. While the initial WCAs are comparable for samples treated in air and oxygen, samples treated in oxygen show significantly longer durations of droplet absorption, probably due to a more evenly and densely functionalized surface.

The highest hydrophobicity has the sample 18, which was treated in oxygen for 90 min and which exhibits the highest WCA and duration of droplet absorption. Therefore, this sample has been subjected to further analyses, including investigation of agglomeration in the mixture with the powdered polypropylene matrix. In the text below it is referred as “functionalized cellulose.”

### 3.2. XPS Analysis

Samples of cellulose have been subjected to XPS analysis. The relative mass fraction of detected carbon and oxygen in the untreated cellulose (zero sample) was 61.1% and 38.9%, respectively. In the case of functionalized cellulose, the relative mass fraction of the detected carbon decreased to 42.5%, while the relative mass fraction of the detected oxygen increased to 46.1%. In addition, significant increase of silicon content has been observed since the silicon relative mass fraction of 11.4% was also detected in the functionalized cellulose. The results are summarized in the Table 2.

**Figure 4:**
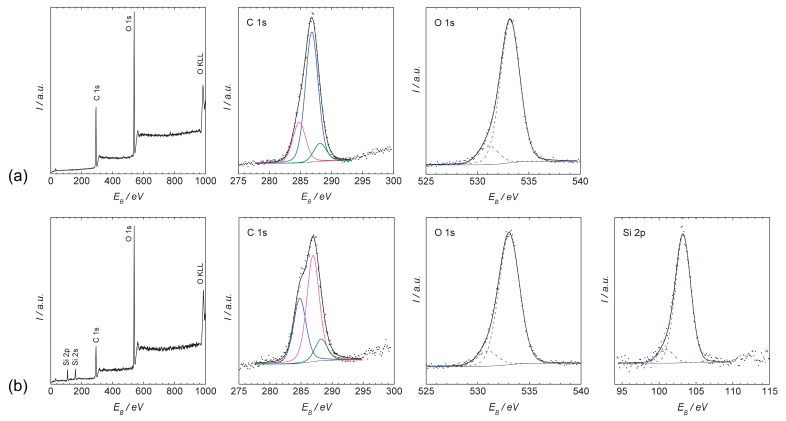
XPS spectra of the (**a**) zero sample and (**b**) functionalized cellulose.

The photoelectron spectra of untreated (zero sample) and functionalized cellulose are shown in the Figure 4. A photoemission line with a binding energy of 100.9 eV and a photoemission line with a binding energy of 103.2 eV can be identified in the spectrum for silicon (Si 2p) of the functionalized cellulose shown in Figure 4b. A photoemission line with a binding energy of 100.9 eV can be assigned to the Si–C chemical bond. Due to the hydrophobicity of the functionalized cellulose and the chemical structure of used precursor (HMDSO), Si–CH_3_ bonds are likely to be present on the surface. Photoemission lines with a binding energy near to 103.2 eV are often assigned to the chemical bond of SiO_2_, SiO_2_C_2_, or SiO_3_C [67,75,76].

The photoelectron spectra of the zero sample and the functionalized cellulose prove significant increase of the proportion of carbon bond (C–H) with a binding energy of 284.8 eV from 21.7% to 33.8% (see Table 3). The Si–C bond has a very close binding energy of approximately 284.6 eV [75]. Thus, a larger carbon bond population may be responsible for the binding of silicon to the surface of the functionalized cellulose [67].

The chemical bonds probably presented on the surface of the functionalized cellulose suggest that the detected chemical structures may resemble some silane coupling agents with organofuctionalities used in natural fiber composites, particularly the VTS coupling agent with vinyl functionality [62].

### 3.3. SEM Analysis

**Figure 5:**
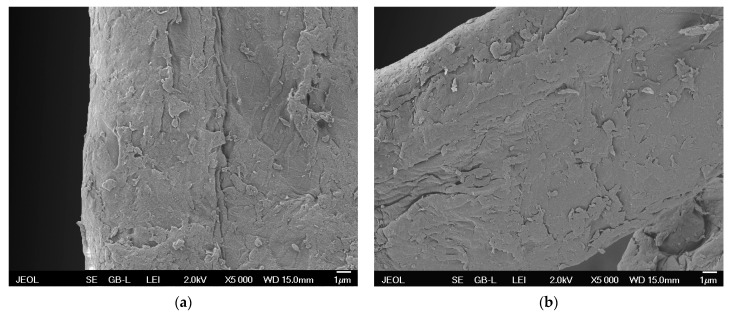
SEM images of (**a**) untreated cellulose fiber (zero sample) and (**b**) functionalized cellulose fiber.

Figure 5a shows SEM image of the untreated cellulose fiber (zero sample), while Figure 5b shows SEM image of the functionalized cellulose fiber. SEM images of the untreated and treated cellulose fibers show that both fibers are not completely smooth on their surface. The differences in surface morphology between these two samples are not observable. Slight difference in the color of both images can be attributed only to the technical quality of the images.

In the SEM images no inhomogeneous or discontinuous films are visible. Therefore, considering the significant change in the surface wettability, the cellulose surface has been very probably functionalized in terms of incorporation of organosilane functional groups into the treated surface.

Moreover, no burrs or other undesirable morphological changes are observable on the fiber surface in the SEM images of treated cellulose, so the plasma treatment does not cause degradation of fiber surface, although the duration of the treatment process is relatively long.

### 3.4. Proposed Mechanism of Cellulose Functionalization by Organosilane Groups

Based on the results of XPS and SEM analysis and considering the optimized process parameters leading to the highest hydrophobization of the substrate, we propose a mechanism of cellulose functionalization by organosilane groups during the treatment in low-pressure microwave plasma discharge.

**Figure 6:**
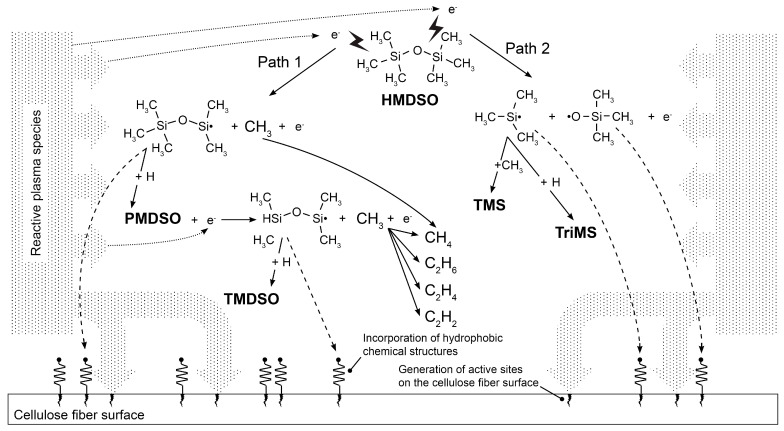
Scheme of the interaction of HMDSO dissociation products with reactive plasma species and cellulose fiber surface during the low-pressure microwave plasma treatment [77].

As can be seen in Figure 6, the chemical conversion of HMDSO is based on the breaking of the molecule into several fragments. Dissociation can start with the impact of plasma electrons that can break the Si–C bond which has the lowest bond-dissociation energy (3.7 eV) of all bonds present in HMDSO molecule. In this case, a relatively large silicon radical and a methyl group are formed. Recombination of these radicals with hydrogen atoms leads to the formation of stable pentamethyldisiloxane (PMDSO). Following reaction of PMDSO with plasma electrons can lead to the formation of another silicon radical and methyl group. This silicon radical can recombine with a hydrogen atom to form tetramethyldisiloxane (TMDSO). The formed methyl groups may further form several hydrocarbon structures such as CH_4_, C_2_H_6_, C_2_H_4_, and C_2_H_2_ [77].

Dissociation of HMDSO molecule can also start by breaking the Si–O bond with a bond-dissociation energy of 4.6 eV. In this case, smaller silicon radicals are formed, which subsequently react with the methyl group and hydrogen atom to form tetramethylsilane (TMS) and trimethylsilane (TriMS), respectively.

All of the HMDSO fragments mentioned above may be involved in plasma processes, however, HMDSO dissociation starting with breaking the Si–C bond seems to be dominant.

Considering that the highest increase in hydrophobicity was observed after plasma treatment in oxygen-containing process gas, oxidation processes with actively involved oxygen appear to play an important role in the formation of hydrophobic functional groups on the cellulose surface. Presented results correspond to our previous studies [66,78,79] and the findings reported in [80] that noble gases, such as argon, show a reduced capability in HMDSO dissociation compared to oxygen.

In addition to electrons, oxygen plasma generates many reactive species, such as reactive oxygen, atomic oxygen (O), ozone (O_3_), superoxide anion (•O_2_^−^), peroxide (•O_2_^−2^). After interaction with hydrogen atoms, hydrogen peroxide (H_2_O_2_), hydroxyl radical (•OH) and hydroxyl ion (OH^−^) can also be formed [81,82]. These reactive species can react with HMDSO fragments, as well as with the formed hydrocarbons.

While the HMDSO molecule is fragmented, reactive species generated in the microwave plasma discharge also affect the cellulose surface by forming the active sites. The reaction of reactive plasma species with HMDSO fragments leads to the incorporation of hydrophobic functional groups probably bonded by Si–C, SiO_2_, SiO_2_C_2_, or SiO_3_C bonds to the active sites on the cellulose surface. A similar incorporation mechanism has also been described in [67].

**Figure 7:**
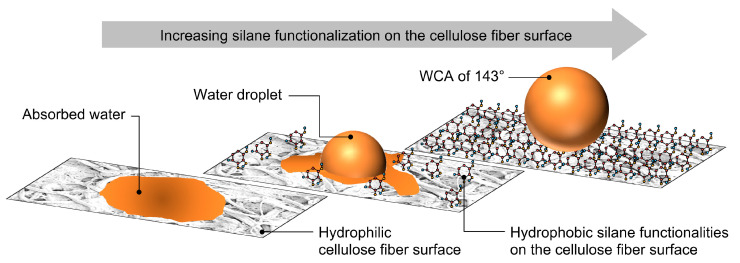
A schematic illustration of silane functionalization of the cellulose fiber surface during the low-pressure microwave plasma treatment in oxygen process gas and its interaction with water droplets.

The observed dependence of the duration of water droplet absorption on the treatment time can be explained by the gradual incorporation of hydrophobic organosilane functional groups to the surface of the cellulose fibers during the treatment process, as shown in Figure 7.

### 3.5. Agglomeration of Cellulose in the Mixture with Polypropylene Powder

Changes in agglomeration of untreated cellulose and functionalized cellulose in the mixture with polypropylene powder after the mixing process have been studied on three different mixtures of polypropylene with cellulose.

The first mixture contained polypropylene and untreated cellulose without pretreatment. The second mixture contained polypropylene and untreated cellulose, which was premixed in a blender for 90 min (corresponding to the plasma treatment time of the functionalized cellulose). The third mixture contained polypropylene and functionalized cellulose.

**Figure 8:**
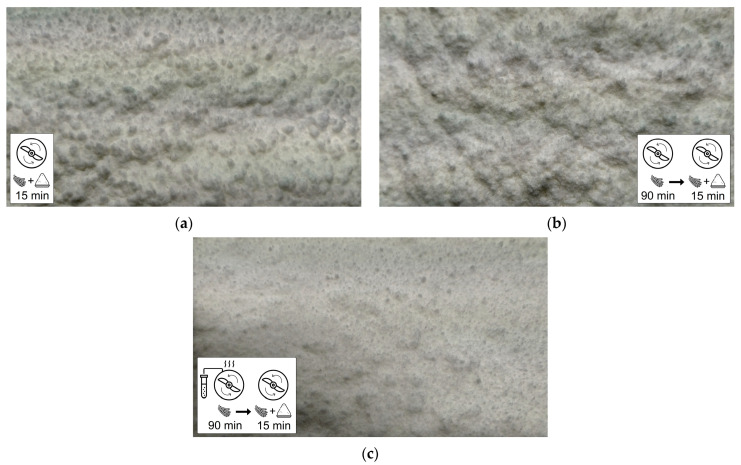
Photographs of the mixtures of polypropylene with three different cellulose samples after the mixing process, where bundles of cellulose fibers are highlighted using High dynamic range (HDR) technology: (**a**) PP mixture with untreated cellulose; (**b**) PP mixture with untreated cellulose mechanically premixed in the blender without plasma treatment for 90 min; (**c**) PP mixture with functionalized cellulose.

Figure 8 presents photographs of prepared mixtures. High dynamic range (HDR) technology was used to highlight the bundles of cellulose fibers. The mixture of polypropylene and untreated cellulose without pretreatment visibly contained large agglomerates despite the mechanical affecting on cellulose fiber bundles during the 15 min mixing process (see the Figure 8a).

In the mixture containing cellulose premixed for 90 min in the blender, the presence of large agglomerates can also be observed as in the case of the mixture with untreated cellulose. However, the bundles of cellulose do not have a visibly spherical shape and the mixture seems to exhibit a slightly higher homogeneity, as shown in Figure 8b.

The third sample of the mixture of polypropylene and functionalized cellulose (Figure 8c) contained only a small amount of much smaller spherical agglomerates and overall exhibited significantly higher homogeneity. Since the mixture with the premixed cellulose contained large agglomerates similar to the mixture with the untreated cellulose without premixing, it can be ruled out that the mechanical mixing process would have a significant effect on the higher homogeneity of the mixture with the functionalized cellulose. Conversely, it can be stated that the higher homogeneity of the mixture with the functionalized cellulose is caused mainly by the absence of functional groups present on the surface of untreated cellulose forming hydrogen bonds between the hydrophilic fibers.

## 4. Conclusions

1. The functionalization process of cellulose fibers by hydrophobic organosilane functional groups in a low-pressure microwave plasma discharge using HMDSO precursor was designed and optimized.
  - The degree of the obtained hydrophobicity strongly depends on both tested process conditions (used process gas and treatment time). With an increase of the treatment time, the density and uniformity of the organosilane functionalization is likely to increase and the treated cellulose fibers become less hydrophilic. Considering that treatment in argon causes only a slight change in wettability, while the treatment in air and oxygen leads to high hydrophobicity of the cellulosic fibers, oxygen appears to be responsible for hydrophobizing the cellulose fiber surface through the incorporation of organosilane functional groups.
  - The application of oxygen/HMDSO microwave low-pressure plasma discharge enabled the creation of highly hydrophobic cellulose fibers with initial WCA up to 143° and the duration of droplet absorption up to 229 s.
2. The functionalized hydrophobic cellulose was subjected to analyses of chemical composition and surface morphology.
  - XPS analysis confirmed the presence of silicon in Si–C, SiO_2_, SiO_2_C_2_, and SiO_3_C bonds on the surface of the functionalized cellulose.
  - Based on the SEM analysis, the treated cellulose fibers do not exhibit degradation or any differences in surface morphology compared to the untreated cellulose fibers. The absence of a visible deposited film or other deposited structures on the surface indicates that the treated cellulose fibers have been functionalized with organosilane functional groups.
3. The hydrophobic organosilane functionalized cellulose exhibited visibly lower tendency to agglomerate in the significantly more homogeneous mixture with non-polar powdered polypropylene matrix.
4. With respect to the following perspectives, we conclude that cellulose treated with the presented plasma functionalization process represents a promising filler for NFPCs.
  - The lower agglomeration of treated cellulose fibers during the mixing process can contribute to a more even dispersion of the fibers in the non-polar polypropylene matrix potentially leading to better mechanical properties of prepared composites.
  - The hydrophobicity of the treated cellulose fibers can significantly reduce the moisture absorption avoiding the fiber swelling and the reduction of their mechanical properties.
  - Due to its similarity to conventional organosilane coupling agents, the organosilane functionalized surface of treated cellulose fibers may be chemically compatible with the non-polar polypropylene matrix to form covalent bonds with a significant increase of interfacial adhesion.

## Figures and Tables

**Table 1:** Wettability of cellulose samples depending on the process parameters.

Sample No.	Process Gas	Treatment Time (min)	Initial WCA (°)	Duration of Droplet Absorption (s)
Zero sample	-	-	20	0.08
1	argon	15	22	0.08
2		30	23	0.08
3		45	25	0.1
4		60	27	0.15
5		75	28	0.17
6		90	29	0.2
7	air	15	26	0.1
8		30	128	1.3
9		45	136	9.8
10		60	137	15.2
11		75	139	20.4
12		90	139	29.8
13	oxygen	15	110	0.17
14		30	124	0.62
15		45	137	40.2
16		60	138	90.3
17		75	143	171.4
18		90	143	229

**Table 2:** The relative mass fraction of detected elements in the zero sample and in the functionalized cellulose.

Elements (%)	C	O	Si
Zero sample	61.1	38.9	-
Functionalized cellulose	42.5	46.1	11.4

**Table 3:** Population of chemical bonds in the zero sample and in the functionalized cellulose.

Population of Chemical Bonds (%)	C–H	C–O	O–C–O, C=O
Zero sample	21.7	68.9	9.4
Functionalized cellulose	33.8	55.2	11

## Data Availability

Data is contained within the article.
